# Long-range Regulation of Partially Folded Amyloidogenic Peptides

**DOI:** 10.1038/s41598-020-64303-x

**Published:** 2020-05-05

**Authors:** Shayon Bhattacharya, Liang Xu, Damien Thompson

**Affiliations:** Department of Physics, Bernal Institute, University of Limerick, Limerick, V94 T9PX Ireland

**Keywords:** Computational models, Computational chemistry, Molecular dynamics

## Abstract

Neurodegeneration involves abnormal aggregation of intrinsically disordered amyloidogenic peptides (IDPs), usually mediated by hydrophobic protein-protein interactions. There is mounting evidence that formation of α-helical intermediates is an early event during self-assembly of amyloid-β42 (Aβ42) and α-synuclein (αS) IDPs in Alzheimer’s and Parkinson’s disease pathogenesis, respectively. However, the driving force behind on-pathway molecular assembly of partially folded helical monomers into helical oligomers assembly remains unknown. Here, we employ extensive molecular dynamics simulations to sample the helical conformational sub-spaces of monomeric peptides of both Aβ42 and αS. Our computed free energies, population shifts, and dynamic cross-correlation network analyses reveal a common feature of long-range intra-peptide modulation of partial helical folds of the amyloidogenic central hydrophobic domains *via* concerted coupling with their charged terminal tails (N-terminus of Aβ42 and C-terminus of αS). The absence of such inter-domain fluctuations in both fully helical and completely unfolded (disordered) states suggests that long-range coupling regulates the dynamicity of partially folded helices, in both Aβ42 and αS peptides. The inter-domain coupling suggests a form of intra-molecular allosteric regulation of the aggregation trigger in partially folded helical monomers. This approach could be applied to study the broad range of amyloidogenic peptides, which could provide a new path to curbing pathogenic aggregation of partially folded conformers into oligomers, by inhibition of sites far from the hydrophobic core.

## Introduction

Protein conformational disorders including Alzheimer’s (AD) and Parkinson’s disease (PD) present the hallmark features of misfolding, self-assembly, and accumulation of monomeric precursor peptides known as intrinsically disordered proteins (IDPs) or amyloidogenic peptides such as amyloid-β, Aβ (implicated in AD) and α-synuclein, αS (in PD)^1^. Low molecular weight soluble oligomers are thought to be neurotoxic in the self-assembly pathway^2^, and a number of experiments suggest their formation may proceed *via* α-helical oligomeric intermediates^3–5^. Partially folded helical conformers (monomers) have been reported to be on-pathway to fibril formation^6,7^. Consequently, the tendency to form helical oligomers (through helix-helix associations) may be imprinted within the minor populations of aggregation-prone partially folded helical monomers^3,6–8^ that both Aβ and αS display in membrane-free aqueous solution^9,10^, and which arise *via* conformational exchanges between non-aggregating helically folded and completely unfolded states. Formation of partially folded intermediates has also long been correlated with aggregation and is identified as a key step in fibrillation^11,12^. A powerful mathematical model based on kinetic state transitions for Aβ structural evolution during fibrillogenesis has been able to reproduce the experimentally observed helical intermediates^13^.

The energy landscapes of globular protein folding and binding have been well documented^14^. In addition, aggregation has been linked to an intricate balance between folding and binding^15^. Especially, the ruggedness and ‘frustrations’ of an IDP energy landscape have to a large extent been attributed to fluctuating allosteric transitions^16^. Allostery^17^, or regulation at a distance, plays a central role in mediating protein recognition, signal transduction and promiscuous interactions of IDPs^18,19^. Nuclear magnetic resonance (NMR) relaxation measurements demonstrate correlated collective internal dynamics within IDP ensembles^20–23^, where perturbation (such as folding or ligand binding) within one domain may result in a regulatory effect on a distant, but energetically coupled, domain^24,25^. As disorder is a functional advantage for regulation at long-range^26^, conformational shifts may accommodate molecular recognition of IDPs through hydrophobic interactions^27^ and promote folding-induced binding or conformational selections^28^ during self-assembly in many neurodegenerative diseases^29,30^. Thus, the perturbations in the disordered regions of the helical monomers of Aβ42 and αS may also regulate the tendency to bind other helical monomers and form helical oligomers.

It is extremely challenging to identify and measure these subtle variations in the degree of helicity using spectroscopy measurements, due to the conformational diversity in the helical subspaces^8^. Identification of the difference between the aggregation propensities of helical intermediates is usually impossible due to the very short lifetimes of monomer partially folded states. Thus, little is known about what causes partially folded helical Aβ42 and αS to be so aggregation-prone, compared to the fully folded helical or completely unfolded conformations^6^.

**Figure 1:**
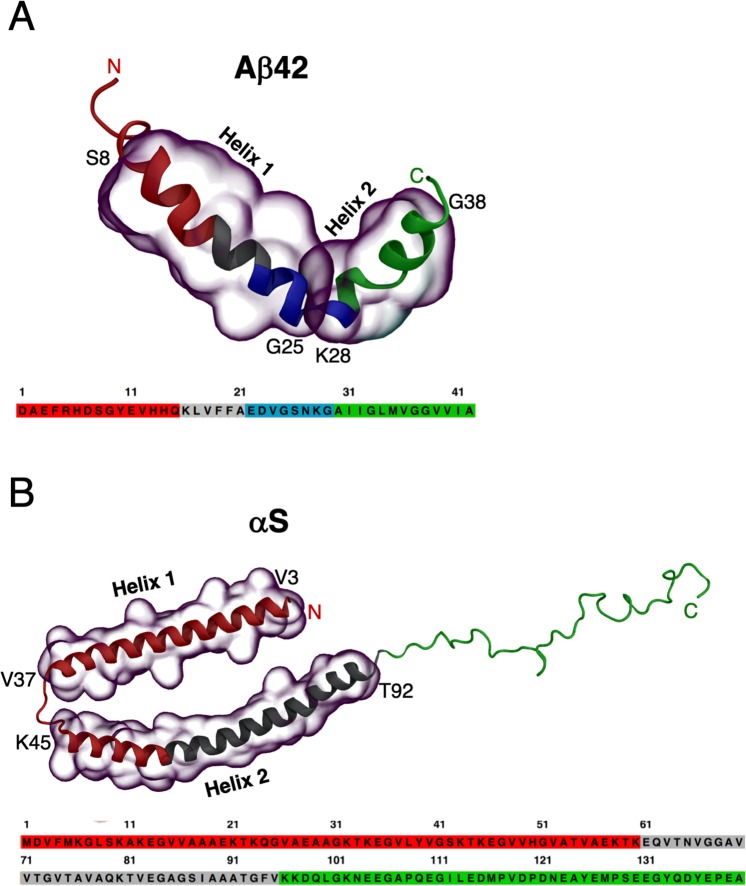
Helix-turn-helix molecular models solved by experimental NMR structures of **(A)** Aβ42 in 20% water/80% deuterated-hexafluoroisopropanol (PDB 1IYT^32^) and **(B)** micelle-bound αS in aqueous solution with sodium dodecyl sulfate (PDB 1XQ8^33^). The amino acid sequences are shown underneath each structure. The regions encompassing Helix 1 and Helix 2 are overlaid with transparent surfaces in both Aβ42 (S8–G25 and K28–G38) and αS (V3–V37 and K45–T92). The colour scheme for the subdomains in the structures and their sequences are, red: N-terminal region, green: C-terminal region, gray: central hydrophobic region (central hydrophobic cluster in Aβ42 or non-amyloid-β component in αS), and blue: turn region.

We have very recently shown from extensive molecular dynamics (MD) simulations that terminal groups in both Aβ42 and αS frequently make direct steric contacts with the central hydrophobic domains that help stabilize folded conformations^31^. Here, we look beyond just direct contacts in folded states and comprehensively map internal long-range communications across helically folded, partially folded and unfolded states of Aβ42 and αS peptides by computing dynamic cross-correlation networks from extensive microseconds-scale MD simulation data. We identify functionally relevant sub-domains involving the charged termini ends that remain 10 − 20 Å from the hydrophobic domains yet play a critical role in modulating their helical folds, through long-range coupling. Starting from solved helix-turn-helix structures of both Aβ42 and αS (see Fig. 1)^32,33^, we employ a range of physical models with diverse helix-coil transition propensities to generate a broad distribution of conformational states (see Methods). No single physical model has been able to produce consistent results for both the folded and the disordered states of IDPs (or the transitions in-between)^30,34–37^, and so we take advantage of model inhomogeneity to sample extensively in the rich IDP conformational sub-spaces. In detail, the traditional CHARMM36^38^ protein model with CHARMM-modified TIP3P water^39^ (TIP3P) and CHARMM36 with TIP4P^40^ parametrized for globular proteins gave as expected predominantly fully-helical folded states while the more IDP-specific CHARMM22*^41^ with TIP4P-D^35^ and Amber ff03ws^34^ with the scaled TIP4P/2005^34,42^, gave partial helical folded states together with some completely unfolded (disordered) states (see Methods). We focus here on characterising the atomic-scale interactions that stabilise helix formation in the smallest, monomeric species, as the simplest starting point in their oligomerisation *via* conformational selection to helical oligomers. The specific scientific question is, what causes partially folded Aβ42 and αS to be so aggregation-prone, compared to the fully folded helical and unfolded conformations? In the present study, we monitor long-range coupling between domains that may influence and possibly direct, helix-helix associations of amyloidogenic peptides.

## Results

### Free energy surfaces identify and characterize partially folded helical states

Initial classification of the helical subspaces was carried out by evaluating the temporal evolution of the model helical propensities (see Fig. S1A under Supplementary Information). We observe that the computed rates of helix unfolding (and thus the complete unfolding time) differ according to the choice of force field/water model, such that a fast unfolding identifies the short-lived partially folded helical states and a slow unfolding sample long-lived partially folded helical state. This enabled us to generate an extensive conformational ensemble of helical states for both Aβ42 and αS. We compute the helix (α-helix + π-helix + 3_10_-helix) unfolding patterns and the corresponding population distributions of Aβ42 and αS from 2D free energy maps using the residual helical percentages and radius of gyration (R_g_) as order parameters. The convergence of the equilibrium MD simulations (EMD) was assessed by the time cumulative average helical content (Fig. S1B). We also performed Hamiltonian replica exchange simulations with solute scaling (H-REST; see Methods for details) to gauge the extensiveness of our helical conformational sampling with EMD. Fig. S1C shows significant overlaps of the free energy surfaces (tested for αS with CHARMM22*/TIP4P-D) of partially folded states with EMD and refolded helices from completely disordered states with H-REST confirming the robustness of our EMD simulation. The complete helically folded states in our EMD simulations record a high percentage of helices. While such high helical content events might be extremely rare under physiological conditions in water, we reiterate that the idea here is to use molecular models to sample extensively in this regime to capture differences in helical stability that could be highly consequential for pathology^5,6,43^ but remain imperceptible at experimentally accessible timescales.

**Figure 2:**
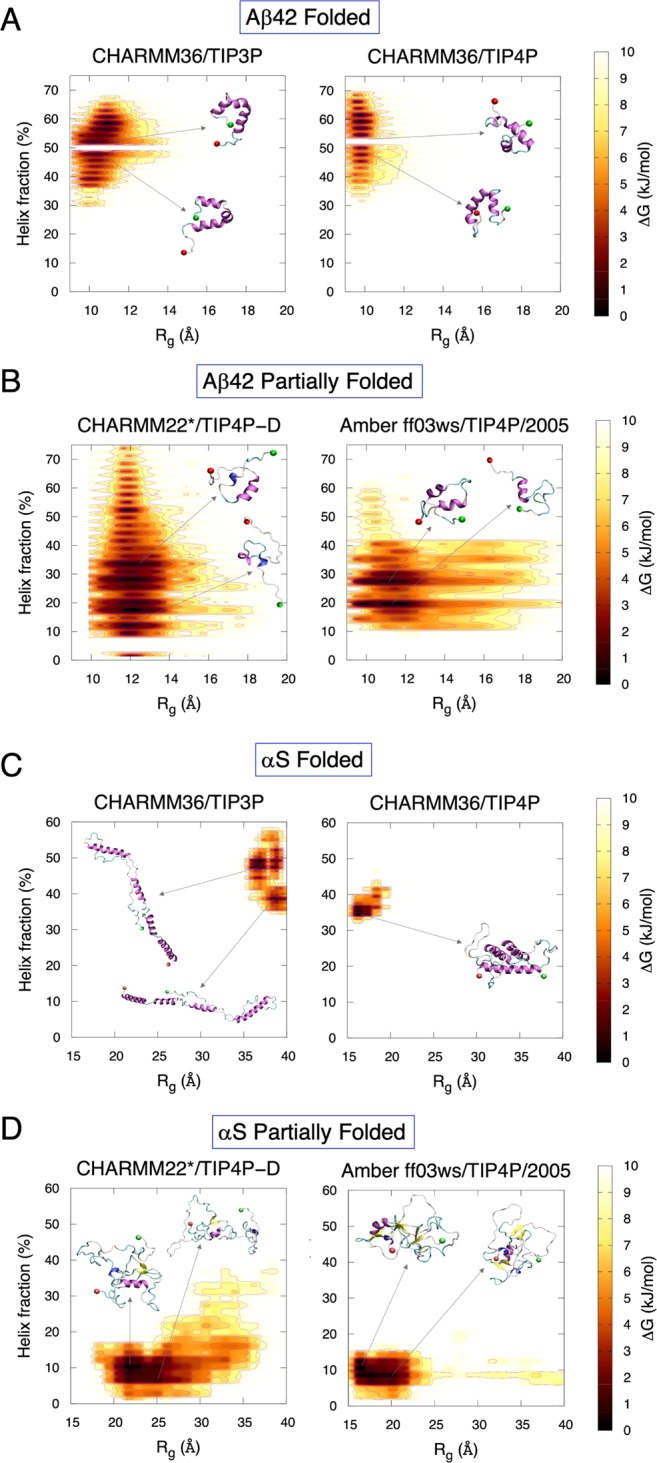
Free energy surfaces of fraction of helix content (%) and radius of gyration (R_g_) for folded (**A**,**C**) and partially folded states (**B**,**D**) of Aβ42 and αS. The representative conformations corresponding to energy minima basins are marked. The N- and the C-terminus are represented as red and green spheres, respectively.

Aβ42 displays dense minima for the helically folded states (Fig. 2A) in the left high-helicity/small-radius energy basin. A sharp energy barrier separates conformations with less than ~50% helical content sampled at low R_g_, which reflects the difficulty in initiating helical unfolding from a predominantly compact state. The MD trajectories show that a helical turn spanning residues G9–V12 in the N-terminal abruptly unfolds causing a sharp drop in overall helicity. This accounts for the energy barrier of ~10 kJ/mol (whitespace) at 50% helicity in the maps, which agrees well with the previous estimate of a free energy barrier of 19 ± 10 kJ/mol from simulated unfolding of two α-helical turns of surfactant-associated polypeptide C^44^. The regions in the Aβ42 peptide can be designated as the N-terminal region (NTR, spanning residues 1–16), central hydrophobic cluster (CHC, 17–21), the turn region (22–29)^45^, and the C-terminal region (CTR, 30–42) (refer to Fig. 1A). The secondary structure (Fig. S2A) shows helix-rich (>80%) distal NTR (residues Y10–K16), full CHC, proximal CTR (A30–G38), and proximal turn region (E22–S26) for the folded states, with no instance of extended structures, while the remaining residues form random coils and turns.

The partially folded helical states populate the minima of low-helicity/large-R_g_ basins (Fig. 2B) as Aβ42 forms a more open structure. In contrast to the folded states, no such sharp energy barrier is observed for the partially folded states. The significantly lowered helix population of partial folds in the distal NTR, CHC and CTR is compensated by turn regions, demonstrating the conformational plasticity (Fig. S2A). More extended structures (β-strand + β-bridge) are extremely rare and transient, with helices being the predominant structural motif.

For αS, stable compact folded helices and elongated helices (Fig. 2C) are both observed covering narrow ranges of small and large R_g_, respectively, any with ~35–60% helicity. Significant barriers identify distinct helical breaks at Gly residues (G31, G36, G67, G68, G73, G84 and G86) leading to transition from a higher helicity (smaller R_g_) to a lower helicity (larger R_g_). The specific regions in the peptide can be identified as the NTR (spanning residues 1–60), the non-amyloid-β component (NAC, 61–95), and the CTR (96–140) (refer to Fig. 1B). Persistent helices in the NTR and distal NAC (V70–V95) can be seen in the folded states of αS (Fig. S2B), with negligible extended structures, except A90 and T92 in the CTR, which forms transient β-strands. The CTR remains mostly random coil, with some small regions forming turn motifs (D98, Q99, E114 and D115), alongside small parts of NTR (K23 and Q24) and NAC (V63–V66, G84, A85, A90 and A91).

The dense population of the partially folded states (Fig. 2D) sit mainly in the lower-left quadrant of the free energy landscape. The R_g_ is densely populated between 15–35 Å, which identifies a heterogeneous population, but is at the same time fairly compact given the length of αS. Sparsely populated large R_g_ basins demonstrate that formation of extended helical states might be a minor event during complete unfolding, and not necessarily a requirement for unfolding helices as was seen for Aβ42. The helical population in the partially folded states is dominant in the NAC region (Fig. S2B). Overall, the unfolding pattern of αS involves a more compact helix unravelling than Aβ42, with low occurrence of helical refolding. Conformational heterogeneity is most prevalent in the partially folded states for both peptides.

The fold propensities of the peptides can also be estimated from their effective dimensions *D*, which is a measure of the distribution of residues over different length scales and can be used to complement the R_g_. The effective dimensions can be obtained by power-law scaling of the structure factor *S(q)* as a function of the wave vector *q*, given by,

1 $$S(q)=\langle \frac{1}{N}|\mathop{\sum }\limits_{j=1}^{N}{e}^{-I\overrightarrow{q}.{r}_{j}}{|}^{2}{\rangle }_{|\overrightarrow{q}|}$$

where |*q* | = 2*π/λ* is the wave vector of wavelength *λ*, and *r*_*j*_ is the residue position. We calculated the variation of the structure factor with wave vector for different fold propensities (Fig. S3), employing the smooth Particle Mesh Ewald summation^46^ method for obtaining high resolution *S(q)* values. Several previous studies have estimated the effective dimension of proteins by employing coarse-grain Monte Carlo (MC) simulations to predict changes in structure factor over a range of temperatures, where the wavelength *λ* was given in MC lattice units^47–49^. Here, we applied the *B*-spline interpolation-based approach for the paired interatomic potential lattice sums generated in our atomistic MD simulations. The scaling exponent of *S(q)* with *q* from *S(q) ∝ q*^*−1/γ*^ can be used to estimate the effective dimension *D*, by fitting the *S(q)* at different *λ* values within the R_g_, such that *D* ≈ *1/γ*. Inset in Fig. S3A shows a rise in the average R_g_ values as Aβ42 unfolds. Estimates of *−1/γ* (annotated alongside plots) reveals *D* ≈ *1.2* for the fully folded state (CHARMM36/TIP3P) and *D* ≈ *1* for the fully unfolded state (CHARMM22*/TIP4P-D), which compares well with the R_g_.

For typical large globular proteins, a value of *D* ≥ 3 is estimated to be a compact structure, whereas *D* ≤ 2 is usually a random coil structure^47–49^. The effective dimension in both cases for Aβ42 is closer to 2 (than 3) suggesting a random coil-like behaviour which may indicate that Aβ42 is too small (only 42 residues) for accurate estimation of residue distributions with various length scales. By contrast, the *S(q) vs. q* plots for the much larger αS (Fig. S3B) shows that the fully folded state (CHARMM36/TIP4P) is extremely compact and globular in nature (*D* ≈ *8*), while the fully unfolded state (Amber 03 ws/TIP4P/2005) is more open, approaching random coil (*D* ≈ *2.7*).

### Charged terminal tails do not interact with the hydrophobic hotspots of aggregation in the partially folded helical states

**Figure 3:**
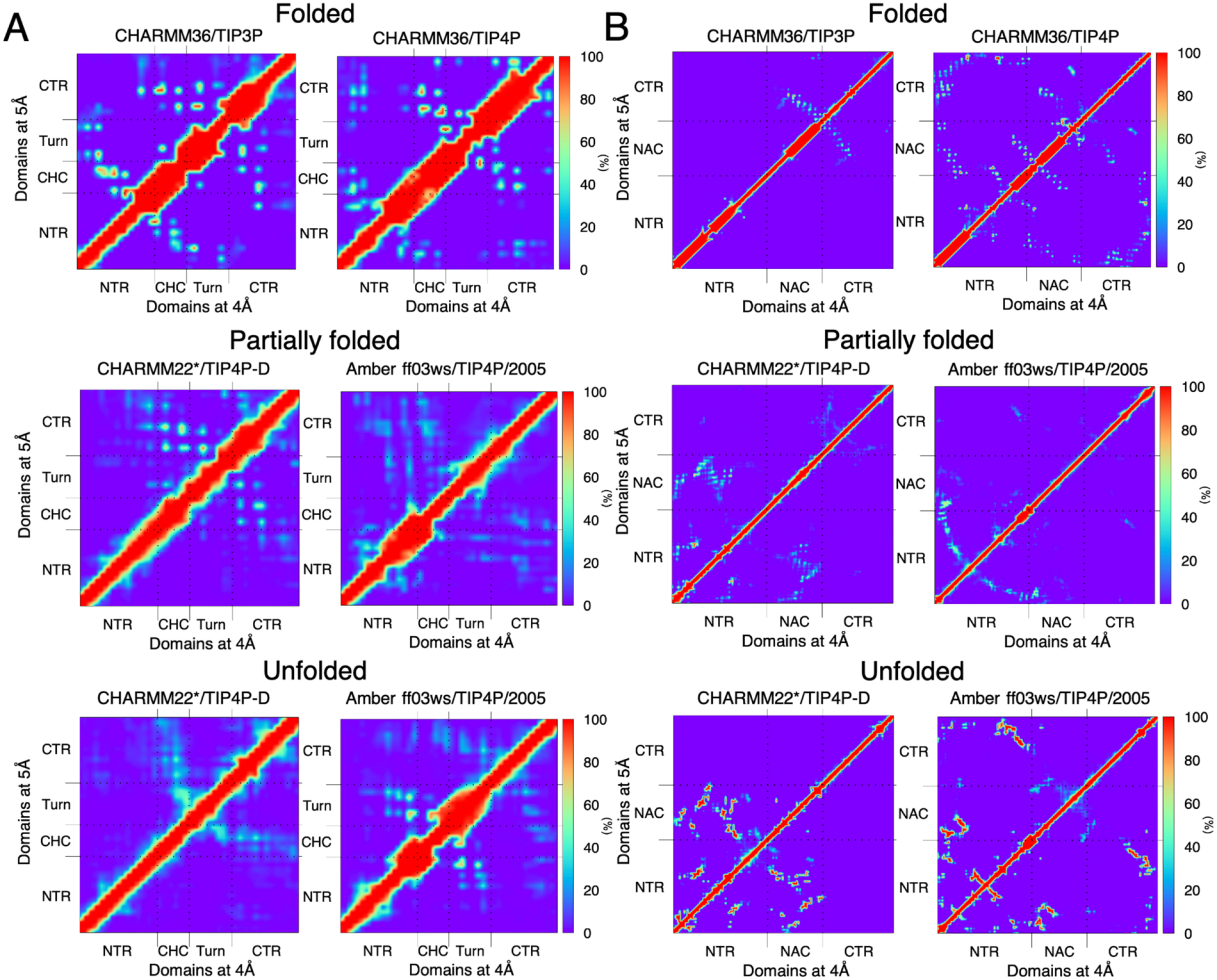
Computed contact maps (frequency, %) between residues in helically folded, partially folded helical, and unfolded states for **(A)** Aβ42, and **(B)** αS. A contact occurs if the minimum average distance between heavy atoms of each residue is within 5 Å (upper left triangle). Contact analyses performed with a 4 Å cut-off (lower right triangle) gave near-identical results. The respective domains in both the peptides are annotated in the map axes.

We have previously demonstrated that the stability of helices in both Aβ42 and αS are governed predominantly by terminal-mediated short-range contacts with the central hydrophobic domains^31^. Here we check if short-range interactions persist also in partially folded helical and unfolded states within a distance of 5 Å. This cut-off distance was chosen in order to accommodate most short-range non-covalent side chain interactions including hydrogen bonds^50^. This cut-off distance was chosen in order to accommodate most short-range non-covalent side chain interactions including hydrogen bonds^50^ and van der Waals forces^51^. Additionally, protein network properties can be robustly evaluated at an optimal intramolecular distance of 5 Å, capturing all important short-range interactions irrespective of changes in force field and/or protein models^52^. We also computed contact frequencies within a tighter cut-off of 4 Å, which may potentially distinguish short-range hydrogen bonds from other non-covalent interactions. Contact probabilities between heavy atoms at a cut-off distance of either 5 or 4 Å across all domain pairs show anti-diagonal contacts (left and right triangles, respectively) for Aβ42 involving the CHC and CTR, corresponding to a helical hairpin-like conformation (Fig. 3A). In addition, frequently sampled contacts (>60%) between the distal NTR (R5–V12) and the CHC are found in the folded states. These contacts are extremely sparse or completely missing in the partially folded helical and unfolded states; instead the CHC-CTR contacts are more prominent. The first four NTR residues are disordered, and do not interact with CHC, but instead with the turn region in the helically folded states. These same interactions are also revealed within the shorter distance cut-off of 4 Å (Fig. 3B, right triangle) indicating no change in contact frequencies from the 5 Å cut-off. In order to identify the dominant force behind these interactions, we computed the interaction types from contacts with frequencies >50%. These interaction maps identify H-bonds, salt bridges and hydrophobic networks between the domains. We show that for all the states of Aβ42, the types of inter-domain interactions are very similar within a cut-off of 5 Å or 4 Å (Fig. S4A). Hydrophobic interactions and salt bridges are the dominant forces for the folded and partially folded states, mainly because of the charged residues in the NTR and the turn region and hydrophobic residues in the CHC. For the unfolded states, we see some remaining H-bonds between the NTR and the turn region.

αS also exhibits anti-diagonal contacts between the domains NAC and the proximal CTR (K96–Q134 away from the CTR tail) in the helically folded states (Fig. 3B), which are sparse (<20%) or totally absent in the partially folded and unfolded states for both 5 Å (left triangle) and 4 Å (right triangle) distance cut-offs. For CHARMM36/TIP4P model, the CTR end (135–140) interactions with NTR, in addition to NTR-NTR and NTR-NAC contacts, stabilize the folded state. By contrast, strong contacts (>70%) between NTR and NAC form in the unfolded states, with the CTR mostly extended into solution. The interaction maps (Fig. S4B) identify predominantly hydrophobic interactions between NAC and CTR, and salt bridge and H-bonds between NTR and CTR for the folded states, and hydrophobic contacts and H-bonds for the partially folded and the unfolded states.

### Cross-correlation maps identify anti-correlated sub-domains that propagate disorder from helical states

**Figure 4:**
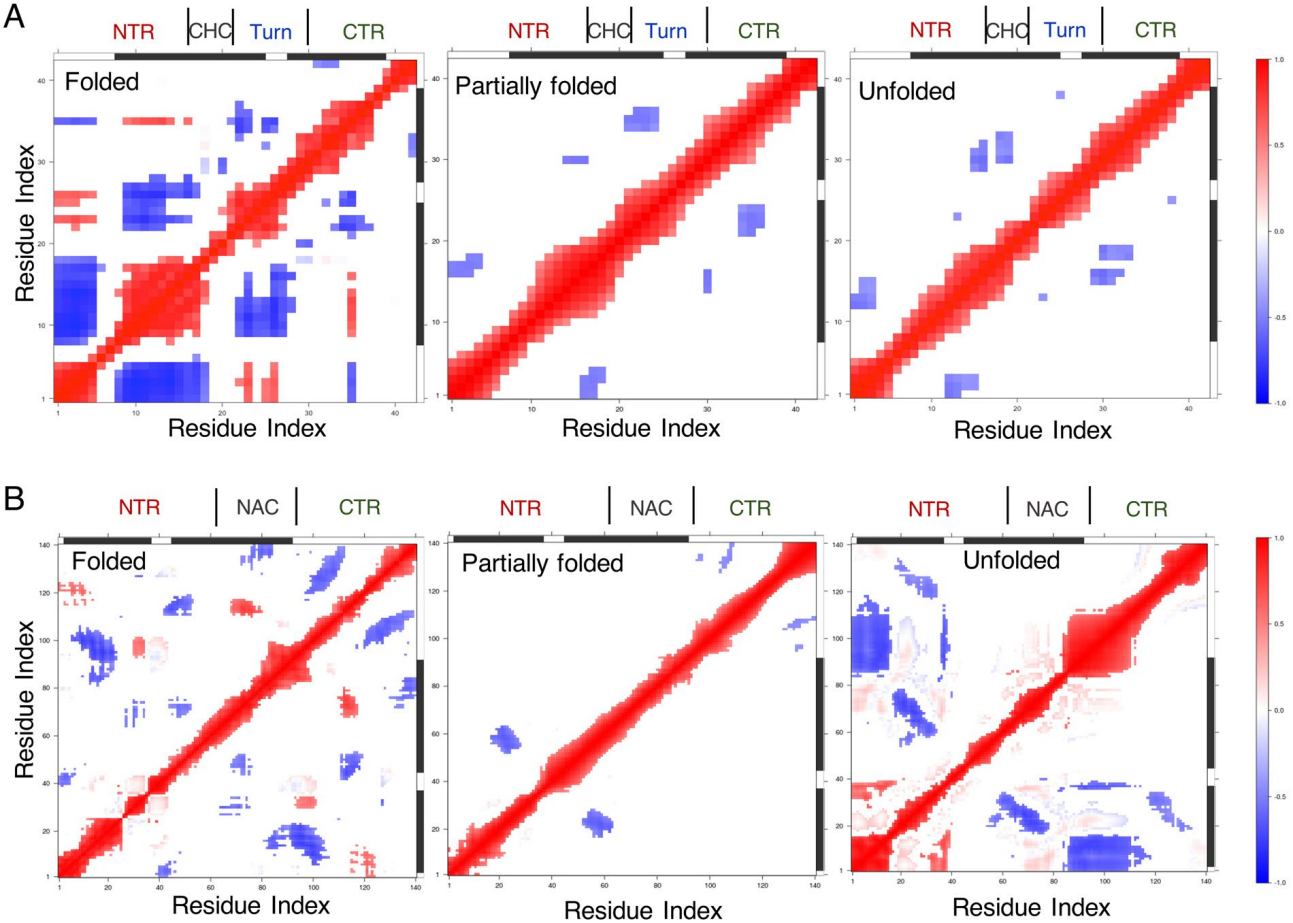
Consensus dynamic cross-correlation maps **(**DCCMs) with a correlation filter of 0.4 and above showing positively (red), negatively (blue) correlated and uncorrelated (white) fluctuating regions for the helically folded, partially folded and unfolded states of (**A**) Aβ42 and (**B**) αS.

To characterize the partially folded states and probe the origin of their higher conformational heterogeneity compared with unfolded states, we performed cross-correlated network analyses of Cα atomic fluctuations using the R package, Bio3D^53,54^ (see Methods) across all states of Aβ42 and αS. Dynamic cross-correlation maps (DCCMs) of Aβ42 filtered at Pearson’s correlation coefficient (PCC or *|c*_*ij*_ | ) ≥0.4 (see Methods for selection criteria of this threshold value) identify positive correlation (red) in the helically folded states between the closely interacting (Fig. 3A) proximal (D1–R5) NTR and the turn region (22–29), and between the distal NTR and M35 in the CTR (Fig. 4A, left panel), which helps preserve the ‘foldedness’ of the helices. However, the overall tendency to unfold helices to random coil (herein referred to as ‘propagation of disorder’ or ‘disorder propagation’) comes from dominant off-diagonal negatively correlated (anti-correlated, blue) sub-domains (cluster of residues within a domain) involving the NTR, turn region and CTR.

The sole cross-correlated regions are actually anti-correlated in the partially folded helical states of Aβ42, which involve communications between the sub-domains of NTR: D1–E3 and CHC: L17–V18, and turn: E22–V24 and CTR: V36–G38, and to a small extent sub-domain of NTR: H14 – K16 and A30 in CTR (Fig. 4A, middle panel). DCCM from completely unfolded states of Aβ42 is further reduced to intra-NTR and CHC-CTR negative correlations (Fig. 4A, right panel). In addition, two off-diagonal weakly anti-correlated residue pairs are observed: one between G25 and G38, and the other between V12 and E22, identifying that these two regions cycle between folding, unfolding and refolding, forming a transient, short β-hairpin motif from the disordered states.

Similar to Aβ42, DCCMs of the folded states of αS retain positively correlated areas at *|c*_*ij*_ | ≥ 0.4 involving close-range (Fig. 3B) couplings between sub-domains of NTR and CTR, and NAC and CTR sub-domains (Fig. 4B, left panel), while a larger number of anti-correlated sub-domains ensure general propensity to unfold helices. As such, the partial folds only retain anti-correlated sub-domains (Fig. 4B, middle panel) encompassing NTR (V15–A27 and V52–K60) and NAC (E61–T64), NAC (F94–K96) and CTR (Y133–D135), and intra-CTR (E104–E110 and S129–A140). Due to the short-range NTR-NAC contacts (Fig. 3B) in the unfolded states, intra-NTR positive correlations are revealed (Fig. 4B, right panel), which are further counteracted by NTR-NAC and NTR-CTR anti-correlations. These off-diagonal NTR-NAC anti-correlated regions (between E20–E35 and K58–A78) identify a β-hairpin region folding from completely disordered states, similar to the anti-correlated residue pairs seen for Aβ42 in the unfolded states. As the purpose of the present work is to characterise the helical ensemble, the potential long-range intra-peptide residue couplings for a monomeric β-hairpin structure will be examined elsewhere. Overall, the general trend of propagation of disorder for both Aβ42 and αS involves anti-correlated motions between sub-domains for all the states.

### Optimal paths of disorder propagation in both helically folded and fully unfolded states skip the central hydrophobic domains

**Figure 5:**
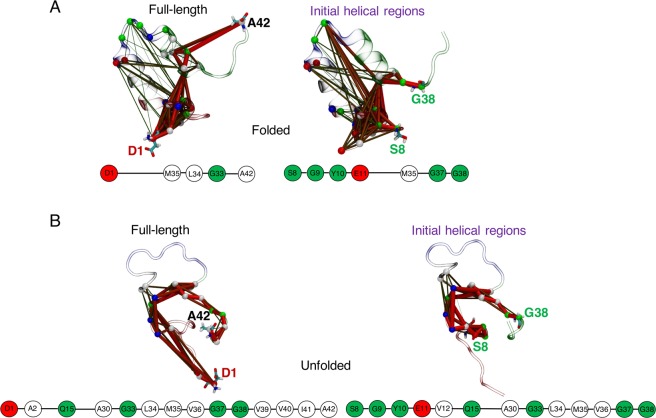
Consensus networks of Aβ42 using a *|c*_*ij*_ | filter of 0.4, showing sub-optimal paths for propagation of disorder from **(A)** helically folded and **(B)** unfolded states, for full-length (source D1 – sink V42) and initial helical regions (source S8 – sink G38) in the folded structure. Network structures are overlaid on representative folded and disordered conformations with domains coloured by region: red = NTR, grey = CHC, blue = turn, and green = CTR. Path thickness reflects inter-residue (intra-domain) coupling strengths. Optimal paths are shown below each network, with residues coloured by hydropathy: white = hydrophobic, green = polar, red = negatively charged, and blue = positively charged. Broken lines between two nodes represent coupling including all residues in between.

Concerted residue fluctuations were modelled by consensus correlation networks with four chosen source-sink residue pairs denoting regions encompassing the full-length peptide, initial helices, helix 1 and helix 2 for both Aβ42 and αS (see Methods) at *|c*_*ij*_ | ≥ 0.4. These consensus networks were computed from combinations of the two physical models (force field and water model) designated for each of the folded, partially folded and completely unfolded states, thus providing robust sampling across both fast (short-lived) and slow (long-lived) unfolding helical states. The path length distributions for the helically folded states of Aβ42 (Fig. S5A under Supplementary Information) show that the overall path of helix 1 is relatively short, while helix 2 path is nearly as long as the initial helical regions. Cooperativity between helices in unfolding is observed as the shortest path of helix 2 (Fig. S5C) from source K28 traces back to S26 (adjacent to end of helix 1) before reaching M35 in the CTR, providing a potential explanation for the high energy barriers faced in unfolding the initial helices of the folded states (Fig. 2A). The optimal path in full-length helically folded Aβ42 (Fig. 5A) shows that disorder propagates through direct coupling of the NTR tip D1 and site M35 of CTR without populating other (sub)domain-specific nodes, which may confer a protective effect on the core helices. A similar trend (path from E11 in NTR to M35 in CTR) is seen when the initial helical regions are considered. Node degeneracy maps (Fig. S5B) reveal M35 forming a centralized hub (Fig. S5D).

The completely unfolded states show non-overlapping path-length distributions, with helix 2 paths being the shortest, but the helix 1 paths are less dense than full-length or initial helical region paths (Fig. S5E). The ideal path on helix 1 skips the CHC region (Fig. S5G, see also minor paths in Fig. S5F and network hubs in Fig. S5H, which mostly involves NTR and CTR), as do the optimal paths of full-length peptide and initial helical regions (Fig. 5B, showing direct coupling between NTR Q15 and CTR A30). However, helix 1 does not include the CTR, which contributes significantly to the path density of the full-length Aβ42 (Fig. 5B).

**Figure 6:**
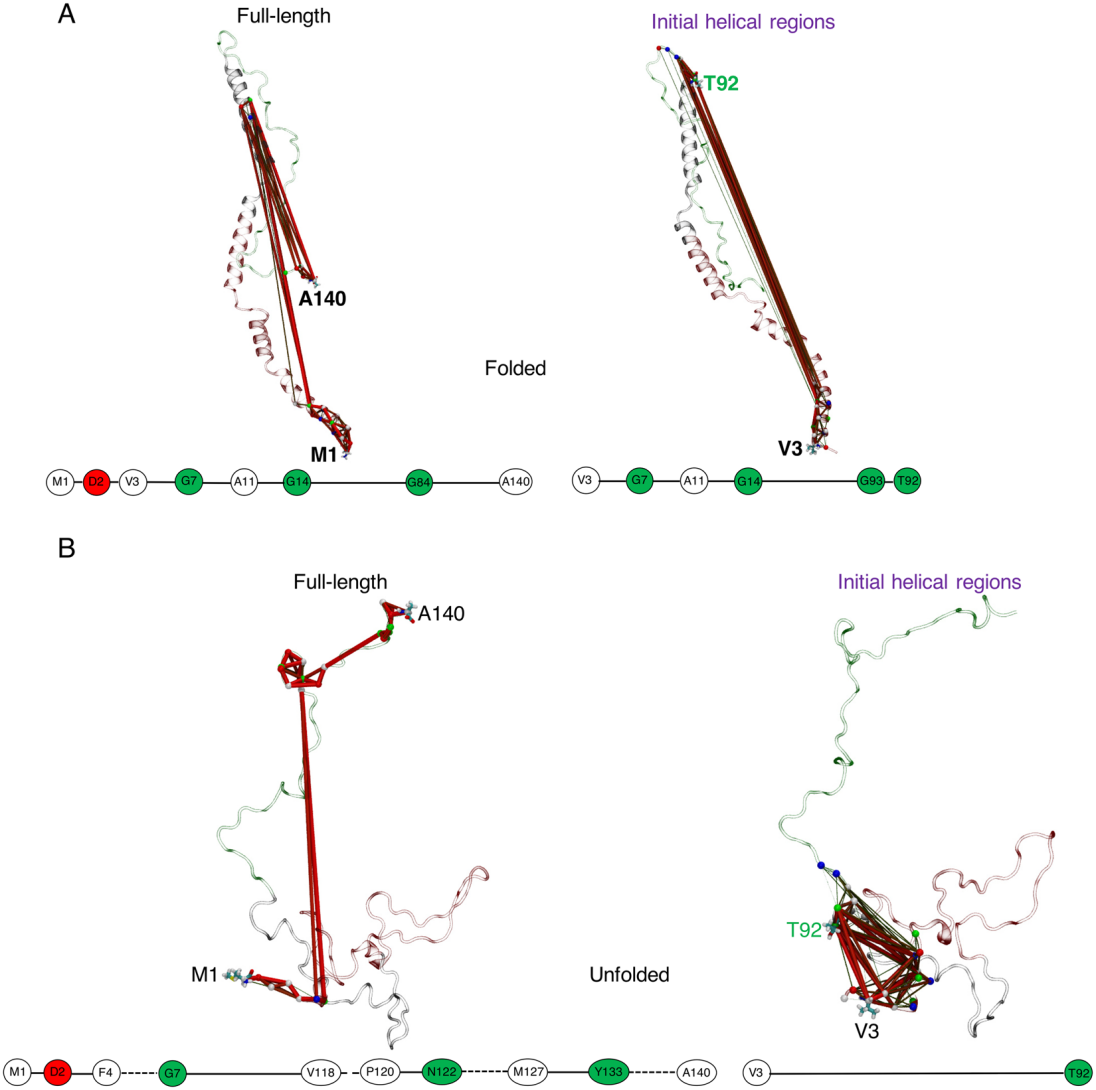
Consensus correlation networks of αS using a *|c*_*ij*_ | filter of 0.4. Network paths for the **(A)** helically folded, and the **(B)** unfolded states. Legend is as for Fig. 5, except domains are coloured as red = NTR, grey = NAC and green = CTR, with different source-sink residue pairs annotating the full-length (source M1 – sink A140) and the initial helical regions (source S8 – sink G38).

In contrast to the Aβ42 CHC region, DCCM of αS (Fig. 4B) shows that sub-domains of NAC are correlated with other regions in folded and unfolded states. However, the optimal paths of disorder propagation in these states (Fig. 6) generally avoid the NAC sub-domains, with only G84 of the NAC falling on the ideal path traversing the NTR (M1 – G14) and CTR for the full-length helically folded states (Fig. 6A). This is because nodes within the NAC that could potentially act as hubs are isolated by long non-populated sequences (Figs. S6D, S6H). For the folded states, the shortest path on helix 1 travels until S42 (close to the start of helix 2) and traces back to the end of helix 1, while for helix 2 it travels all the way back to A18 (belonging to helix 1) before ending with V95 – T92 (Fig. S6C). This indicates a prevalent inter-helical cooperative effect in unfolding the initial stable helices, as also seen in Aβ42. The NTR shows the maximum number of paths (Fig. S6B). The shortest path along the full-length of the disordered states communicates between NTR (M1–G7) and CTR (V118–A140) (Fig. 6B), which for the initial helical regions do not involve any intermediary nodes (direct coupling between V3 and V92; see also Figs. S6E–G).

### Dynamic coupling between terminal tips and central hydrophobic domains confers long-range regulation of partially folded helical states

**Figure 7:**
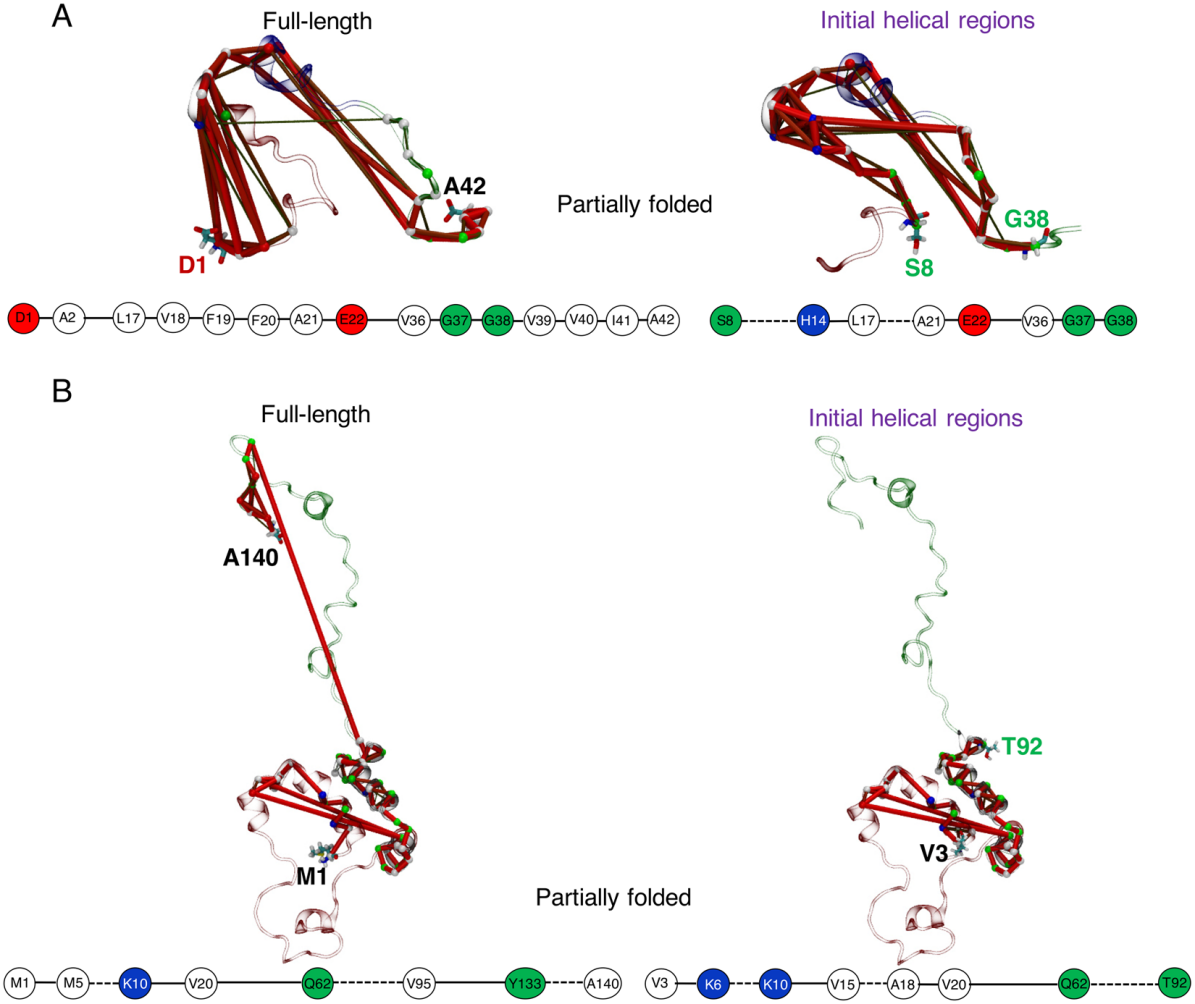
Consensus networks of the partially folded helical states of **(A)** Aβ42, and **(B)** αS following the same scheme of source-sink residue pairs used in Figs. 5, 6.

Networks of partially folded Aβ42 also sample distinct path-length distributions much like unfolded states, but helix 1 paths are densest (Fig. S7A). This is because the CHC region not only traces the shortest path on helix 1 (Fig. S7C), but also allows a greater number of paths to traverse than in the full-length or initial helical paths. The optimal path of disorder propagation from full-length partial helical folds involves couplings between the N-terminal tip (D1–A2) and transiently folded CHC (L17–E22), before terminating at CTR (V36–A42) (Fig. 7A). In the initial helical region, the shortest path skips from the distal sub-domain of NTR (S8–H14) to the CHC region. The paths identify extensive communication between sub-domains of NTR and CHC (Fig. 7A), which do not form short-range (<5 Å) geometrical contacts in the partially folded states. Thus, the CHC residues are extremely degenerate with all source-sink paired paths (except helix 2, which does not include the CHC region) (Figs. S7B, S7D).

In contrast to folded and unfolded states, the optimal path of partially folded states of αS reveal significant coupling between the full NAC domain (Q62–V95) and CTR sub-domain (Y133–A140) spanning the source-sink pair of full-length αS (Fig. 7B). The shortest path for helix 1 identifies similar node connectivity when calculated across full-length or initial helices in the NTR, which for helix 2 is continuous from K45 to V82 mapping the KTKEGV imperfect repeats (Fig. S7G). The path lengths are distributed according to the size of the fragments and are very distinct (Fig. S7E). The dense population of several paths crossing the NAC residues (Fig. S7B) clearly distinguishes partially folded states from the node degeneracy of helically folded or unfolded states (Fig. S6B, S6F) with large gaps on NAC nodes. Finally, the NAC domain is the most prominent network hub, followed by the CTR (Fig. S7H).

### Long-range coupling between charged terminal ends and central hydrophobic domains are modulated at distances up to 20 Å

**Figure 8:**
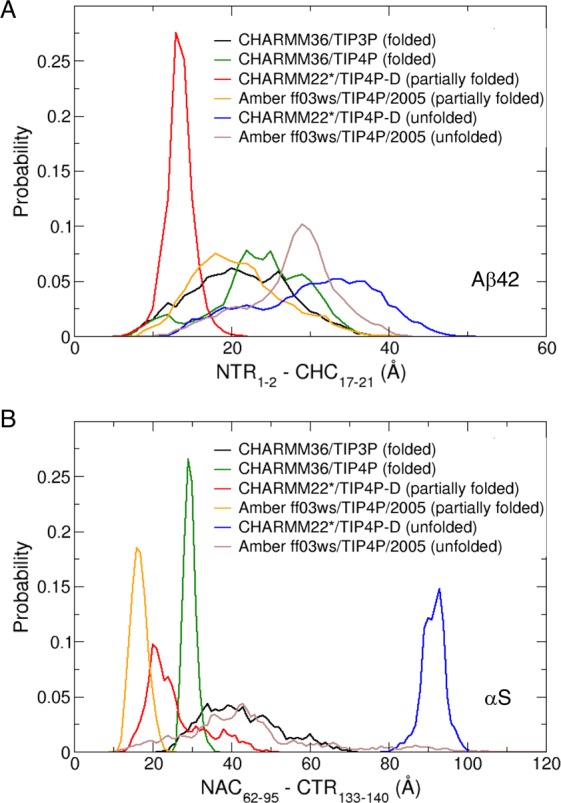
Probability distribution of distances from the **(A)** centre of mass (COM) of D1 – A2 in the NTR or NTR_1-2_ to the COM of the hydrophobic CHC (L17 – A21) or CHC_17-21_ for Aβ42, and **(B)** from the COM of the hydrophobic NAC (Q62 – V95) or NAC_62-95_ to the COM of Y133 – A140 in the CTR or CTR_133-140_ of αS, for folded, partially folded and unfolded states. These regions are shown to be highly correlated in the partially folded helical states (Fig. 7).

We calculated separations between sub-domains involved in concerted long-range coupling in the partially folded helical states, by monitoring the distribution of distances from the centre of mass (COM) of the N-terminal end (D1–A2) of Aβ42 to the COM of CHC (L17–A21), and the COM of C-terminal end (Y133–A140) of αS to the COM of NAC (Q62–V95) (Fig. 8). For Aβ42, concerted fluctuations peak at separations of ~15–18 Å (Fig. 8A) in partially folded states, and for αS at ~18–20 Å (Fig. 8B). By contrast, all helically folded and unfolded states sample longer and/or broader distributions, which indicates that partially folded helical states are modulated by the (predominantly disordered) charged termini tails through long-range couplings up to a distance of 20 Å, beyond which the long-range effect fades out. Sharper peaks in distance distributions within the partially folded helical states of αS identifies that the disordered CTR in αS is able to regulate a comparatively more stable and longer helical NAC with low fluctuations in NAC-CTR distance distributions than shorter and less stable helical CHC, and fluctuations in NTR-CHC distances of Aβ42. It was previously shown that the β-hairpin formed by the Aβ(17–34) fragment is allosterically stabilized by the N-terminal around 10 Å from the centre of the fragment, by the release of entropy to the surrounding water^55^. Our analyses of water dynamics within 10 Å of the most hydrophobic CHC (Fig. S8A) and the fibril-forming core of NAC (68–78)^56^ (Fig. S8B) show subtle differences in temporal water retention between states. Evidently, the cores of the partially folded structures retain more water molecules than either of the disordered or the folded states for both IDPs, which supports the hypothesis that entropically favourable release of retained water may drive inter-molecular oligomeric associations^57^. Note that direct modelling of oligomerization remains extremely challenging even with high-performance computing due to the degeneracy of the free energy surfaces for assembly.

## Discussion

In light of the experimental evidences that early oligomerization of some amyloidogenic IDPs may be linked to formation of helical intermediates by helix-helix associations^3,43,58^, we have extensively modelled the helical subspaces of Aβ42 and αS in order to account for the driving force behind aggregation-prone nature of partially folded helical states, and compared them to helically folded and unfolded states. The sampling strategy employed here combines two different force fields/water models for each of the three states, where differences in helix unfolding rates are used to sample short-lived and long-lived conformations, thus generating an expansive ensemble of folded, partially folded and unfolded states pertinent to the cellular environment. However, the possible existence of other physiologically relevant states cannot be ruled out. For example, both the helical and disordered states sampling efficiency could further be evaluated with the recently parametrized Amber ff99SB-disp^59^ force field, as it is primarily developed to sample both folded and disordered proteins accurately. Many factors will modulate the helical sampling space in a cellular milieu^60^. For example, in a membrane-mimicking environment, Aβ42 has shown predominance of monomeric folded α-helical structures, which resist toxic aggregation^61,62^. The composition of the lipid membrane may also influence the rate of Aβ42 fibrillation, as the oligomers are rich in β-sheet content^63,64^. On the other hand, αS may sample several different helical states on binding to lipid membranes, which could further their aggregation to fibrils^65^. Both extended and broken helical structures have been reported for monomeric αS interacting with phospholipid bilayers^66,67^. Acidic pH may in general facilitate aggregation of amyloid peptides^60^. The conformational ensembles of αS monomers have been shown to form more compact and ordered structures at low pH^68^, while Aβ42 may sample α-helical structures in the acidic environment^69^. Other conditions such as ionic strength and metal ion concentrations, and temperature may have an impact on aggregation propensity and the helical subspace sampling, as summarized in a very recent comprehensive review^60^. The present work focusses on helical conformational sampling monomers in their native state in aqueous solution, which the chosen model components and parameters mimic as closely as possible.

In our previous work, we have highlighted on the role of short-range intra-peptide modulation of the central hydrophobic domains by the flexible termini in stabilizing their aggregation-impeding folded helical states^31^. In this work, we provide an analytical insight into the regulation of aggregation-competent Aβ42 and αS partial helical folds *via* dynamic control of long-range intra-peptide signalling between two distal sub-domains encompassing the charged termini (C-terminus in αS and N-terminus in Aβ42) and the central hydrophobic hotspots of amyloidogenesis.

Free energy maps show that the partially folded helical conformers (intermediate between folded and completely disordered states) in both Aβ42 and αS display a relatively flat surface that samples heterogeneously interconverting states with expansive populations in comparison to the folded helical or unfolded conformations. Contact maps identify that the charged terminal (CT) tips in both peptides do not make short-range (≤5 Å or ≤4 Å) contacts with the central hydrophobic domains (CHD) in the partially folded helical states. However, the dynamic cross-correlation maps reveal that sub-domains of CT and CHD frequently partake in long distance anti-correlated motions, which are absent in unfolded states and counterbalanced by positively correlated sub-domains in the folded states. Computed cross-correlated network analyses of both helically folded and unfolded states reveal that the optimal paths of disorder propagation do not traverse through the CHD in both Aβ42 and αS, but display long-range dynamic coupling between the termini and CHD in the partially folded helical states, conferring that the flexible termini may regulate the local helical folds in CHD.

**Figure 9:**
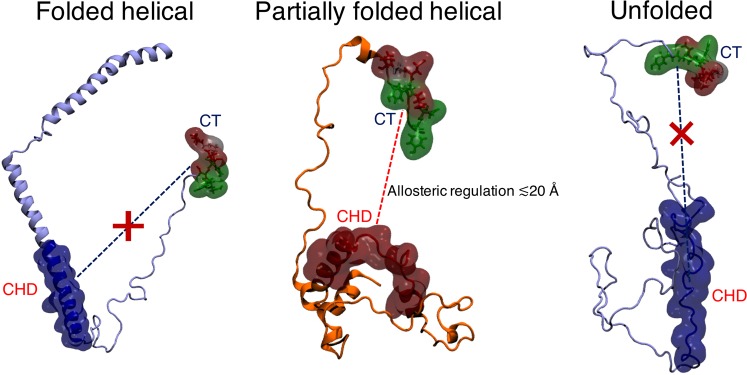
Schematic of proposed mechanism of long-range (up to ~20 Å) regulation between the tip of the charged terminus (CT) and the central hydrophobic domain (CHD) of aggregation-prone partially folded helical states. This coupling is lost at distances >20 Å and is absent for the non-aggregating helically folded and the unfolded states.

Our calculations reveal that the susceptibility to form partially folded helices in the CHD is upregulated by long-range coupling with charged residues in predominantly disordered terminal ends of Aβ42 and αS. This long-range regulatory effect manifests across a distance of up to ~20 Å and might play a major role in promoting helix-helix associations. Our predictive model is consistent with hydrophobic protein self-assembly^70^ modulated by immobilized ions^71^. Hydrophobic interactions can be switched on or off according to spatial arrangements of charged groups^72^, as indicated also from our analyses, providing a potential explanation for why partial helices may be more prone to oligomerization^6,7,43,73^ than predominantly folded helical or unfolded states (Fig. 9). This long-range effect is reminiscent of allosterically coupled sites that may facilitate initial assembly of the peptides. In turn, our predictive models are in strong agreement with the proposed reaction pathways and state parameters of initial aggregation, such that increasing concentrations of solvent-induced α-helical content leads to increased rate of fibrillation up to a certain fraction of helical content (partially folded helices), beyond which the rate of fibrillation slows down considerably (fully folded helices)^13^.

In a more recent study involving an engineered coiled-coil helical dodecamer (12-mer) of the small model peptide ccβ^74^, the same authors used extensive REMD simulations to map the intermediate steps for helix → β-hairpin/β-strand → β-sheet transition during the formation of amyloid-like protofibrils. The identified transitions involved thermally induced conformational straightening of the β-hairpin structures facilitated by breaking of intrapeptide H-bonds, and consequent formation of interpeptide H-bonds with other β-strands to form β-sheets. Furthermore, the authors highlight that the chain additions are a highly cooperative process, and that hydrophobic interactions may play a secondary role in directing the site-specificity of the aligned assemblies. Our predictive models confirm the importance of swapping between intra and inter peptide contacts during structural transitions, involving rearrangements in both H-bond patterns and hydrophobic contacts. Our analysis of helix-helix associations identifies that aggregation may be triggered by decay of intrapeptide H-bonds as the peptides transition from fully to partially folded helices, and also that interpeptide hydrophobic interactions could be modulated by charged terminal residues, which may change the aggregation pathway.

Moreover, our predictions are consistent with those drawn from a master-equation based formalism that was used in conjunction with REMD simulations to map kinetics of dimer formation in the short amyloidogenic peptide NNQQ^75^. Using monomer-monomer closest contact distances as the reaction coordinate, the authors characterized different binding modes that could potentially distinguish fast interconverting binding that forms unstable encounter complexes from slow binding that creates stable dimers. Along similar lines, our analyses reveal that the diversely distributed population of partially folded helical states may form stable helical dimers when there is concerted coupling between the CT and CHD only at distances ≤20 Å. The long range coupling effect is lost above this distance, and may lead to just formation of encounter complexes, in the terminology of ref. ^75^.

There is mounting evidence that the N-terminus of Aβ oligomers are implicated in neurotoxicity^76–78^ and should be considered as promising targets for treatment of AD^79^. In addition to the N-terminus in Aβ42, our unprecedentedly detailed models suggest that the C-terminus in αS may be considered an important target in PD. A feature of the dynamic cross-correlated network analyses method is the difficulty in picking up signals of correlated motions that may be functionally relevant, but which may appear only fleetingly in MD simulations. Attempts made to circumvent this limitation includes use of a Bayesian-based pattern recognition technique named multi-modal DCC^80^. Future tests of this method on intra-peptide long-range communication in IDPs could be useful. In turn, other computational methods for detecting pathways from conformational ensembles generated from MD simulations would include (but are not limited to) normal mode analyses, perturbation-response scanning method, and mutual information analyses^81^. In addition, the information theory measure of entropy transfer can be effectively employed to complement dynamically coupled pathways between residue pairs or domains forming the source-sink pairs in order to assess the causality of correlated fluctuations^82^. Recent works have estimated transfer entropy from MD data to identify driver-responder residues that are involved in unfolding and refolding of α-helix to β-sheet in C-terminal domain of transcription antiterminator RfaH^83^, helix unfolding in Ets-1 protein following binding to the target DNA sequence^84^, folding/unfolding of small proteins^85^, and allosteric activity that governs recognition of Ubiquitin in Ubiquitin-Proteasome binding^86^. We wish to emphasize that a very large computational effort would be necessary to explicitly model the multitude of paths for self-assembly of multiple helical monomers into oligomers – our current dataset provide the required and representative populations of different types of starting helical conformations for such future studies that may become more feasible with advances in high-performance computing and advanced sampling methods. We envisage that the modelling strategy employed in the present study of Aβ42 and αS could be applied to study monomeric helical intermediates of other IDPs^87^ and help find ways to rationally reduce their tendency to promote helical oligomerization^43^.

## Methods

### Molecular dynamics simulations

The initial structures for equilibrium atomistic molecular dynamics (MD) simulations were obtained from the NMR-derived helix-kink-helix structures of Aβ42 solved in 20% water/80% deuterated hexafluoro isopropanol (PDB code 1IYT^32^) and micelle bound human αS solved in aqueous solution with sodium dodecyl sulphate (PDB code 1XQ8^33^). We wanted to monitor the unfolding of helices, so we chose the aforementioned experimental models with predominant helical conformations. We note that other experimental helical structures of Aβ42 (PDB ID: 1Z0Q^88^) and αS (PDB ID: 2KKW^89^) exist. However, 1Z0Q has lower helical content than that of 1IYT, and 2KKW has similar helical regions to 1XQ8, thus explaining our rationale for selecting the helical models as our starting geometry. The two helices for Aβ42 span Ser8–Gly25 (helix 1) and Lys28–Gly38 (helix 2), while for αS they span Val3–Val37 (helix 1) and Lys45–Tyr92 (helix 2). All MD simulations were performed using the Gromacs 5.1.4^90^ package. The starting structures were represented by three different protein force fields – CHARMM36^38^, CHARMM22*^41^ and Amber ff03ws^34^, and solvated in boxes containing four different water models – CHARMM-modified TIP3P^39^, TIP4P, protein-water interaction scaled TIP4P/2005^34,42^ and TIP4P-D^35^ resulting in four distinct combinations of force fields and water models: CHARMM36/TIP3P, CHARMM36/TIP4P, CHARMM22*/TIP4P-D and Amber ff03ws/TIP4P/2005. Additionally, we performed 3.4 μs of Hamiltonian replica exchange (70 ns * 48 replicas simulated at different Hamiltonians). These simulations used solute tempering (scaling)^91,92^ (H-REST) with CHARMM22*/TIP4P-D (αS) starting from a completely disordered state. The steady states of the equilibrium MD (EMD) and H-REST simulations were assessed by the convergence of temporal cumulative average helix percentages (Fig. S1B under Supplementary Information). The converged helical content for CHARMM22*/TIP4P-D with αS for both EMD and H-REST show steady state populations of ~20%, indicating similar helical subspace sampling for the two different methods. The last 200 ns was used for analysis of the EMD simulations of different states (folded, partially folded and unfolded), while the last 20 ns (out of the 70 ns/replica) was used for analysis of the H-REMD simulation. We show using free energy maps of percent helicity and radius of gyration, that the conformational spaces explored for the partially folded helical states in our EMD simulations matches up with H-REST simulations. H-REST sampled conformational spaces of transiently formed helices that are refolded from a completely disordered state, confirming that our sampling of helical subspaces is statistically robust (Fig. S1C).

Physiological concentrations (0.15 M) of NaCl were added to neutralize the peptide formal charge. Na^+^ counterions were added to neutralize the peptide formal charge and 0.15 M of NaCl was added to mimic physiological ionic strength. 3 Na^+^ counter-ions and 25 NaCl ion pairs were added for Aβ42, while 9 Na^+^ counter-ions and 133 NaCl ion pairs were added for the more highly-charged and larger αS simulation cell. All Histidine residues (H6, H13 and H14 in Aβ42, and H50 in αS) were modelled in their neutral states with the ε-nitrogen protonated, as deduced from His contacts to neighbouring residues in the starting structures. Both termini of the two monomers were kept charged. The minimum distance between a protein atom and the boundary of the boxes was set at 20 Å. The protein and water molecule bond lengths to hydrogen were constrained using the LINCS^93^ and the SETTLE^94^ algorithms, respectively. An integration step of 2 fs was chosen for running the simulations employing the velocity Verlet integrator^95^, and coordinates were saved every 20 ps. Long-range electrostatics were treated by the Particle mesh Ewald (PME) method^96^. All systems were minimized for 5000 steps, and equilibrated for 1 ns in NVT ensemble followed by another 1 ns of NPT equilibration with the reference pressure at 1 bar and a time constant of 4 ps using the Berendsen barostat^97^. Protein and non-protein (water and ions) molecules were coupled separately to an external heat bath with a coupling time constant of 1 ps using the velocity rescaling method^98^. The production runs were carried out in the constant pressure and temperature NPT ensemble.

Simulations were run for ~2 μs for Aβ42 and ~1.5 μs for αS with each of the four combinations of protein force fields and waters models (for details of these simulations, see Table 1), amounting to a total run time of ~14 μs. For simulations using CHARMM36/TIP3P and CHARMM36/TIP4P, complete unfolding of the helices does not occur within the simulation time, with both Aβ42 and αS, retaining ~1/3 helicity (α-helix + π-helix + 3_10_-helix, Fig. S1A). These states are hence regarded as folded states. For CHARMM22*/TIP4P-D, fast and complete unfolding occurred by ~0.2 μs for Aβ42 and ~0.6 μs for αS, while for Amber ff03ws/TIP4P/2005 a slower complete unfolding occurred by ~1.5 μs for Aβ42 and ~1.2 μs for αS (Fig. S1A). Hence the last 200 ns before complete unfolding of CHARMM22*/TIP4P-D and Amber ff03ws/TIP4P/2005 are regarded as partially folded states, and the final 200 ns of the trajectories with CHARMM22*/TIP4P-D and Amber ff03ws/TIP4P/2005 are regarded as completely unfolded states. All analyses were carried out using the Gromacs 5.1.4 analysis tools. Interdomain contact probability maps and interaction maps were generated using the CONAN^99^ contact analysis tool and water dynamics was monitored using VMD^100^ software.

**Table 1:** Details of equilibrium MD simulations (EMD) for different states of Aβ42 and αS.

States	Simulation time (ns)	Complete unfolding time (ns)	α-helical content (%mean ± SD)
**Aβ42**			
Folded (CHARMM36/TIP3P)	2014	N/A	50 ± 4
Folded (CHARMM36/TIP4P)	2018	N/A	52 ± 4
Partially folded and Unfolded (CHARMM22*/TIP4P-D)	2011	206	18 ± 6
Partially folded and Unfolded (Amber ff03ws/TIP4P/2005)	2020	1527	20 ± 4
**αS**			
Folded (CHARMM36/TIP3P)	1540	N/A	44 ± 8
Folded (CHARMM36/TIP4P)	1411	N/A	37 ± 5
Partially folded and Unfolded (CHARMM22*/TIP4P-D)	1413	625	8 ± 6
Partially folded and Unfolded (Amber ff03ws/TIP4P/2005)	1488	1160	6 ± 6

### Free energy calculations

The values for the Gibbs free energy (kcal/mol) using two different order parameters were obtained using the equation:

2 $$\Delta G=-{k}_{B}.T.(ln{P}_{i}-ln{P}_{max})$$

where *P*_*i*_ is the probability distribution for pairs of order parameters, and *P*_*max*_ is its maximum, such that *lnP*_*i*_ *– lnP*_*max*_ identifies the lowest free energy point at Δ*G* = 0.

### Dynamic cross-correlation network analysis

Dynamic network analyses of cross-correlated motions were performed using the Bio3D R package^53,54^. Correlated residue pairs were identified using linear Cα atomic displacements, as opposed to linear mutual information^101^. Consensus networks were generated for each of the designated folded, partially folded, and unfolded states of WT Aβ42 and αS, using the method similar to Luthey-Schulten^102^, but with network edges weighted based on the Cα-Cα cross-correlation values to include long-range interactions between residues that are not in direct contact. In a mass-weighted network graph, the residues represent nodes *i* and *j*, and the weights represent the edges with Pearson’s cross-correlated (PCC) values^103^ *c*_*ij*_ connecting the nodes.

A community is defined as a cluster of nodes (residues) that are highly connected through correlated motions. The value of *c*_*ij*_ ranges between −1 and 1. *c*_*ij*_ is closer to −1 when the two coupled residues are negatively correlated or anti-correlated, is 0 when they uncorrelated, and closer to 1 when they are positively correlated. All networks were constructed with a *|c*_*ij*_ | ≥ 0.4 (the modulus represents absolute value taken for both positive and negative correlations) based on the following criteria: *(1)* False positives or false negatives using too low or high values of *|c*_*ij*_ | , respectively, could be circumvented by using a near-midpoint value of cross-correlated PCC. *(2)* The network structure remains consistent between 0.35, 0.4 and 0.45 allowing similar community partitioning. Dynamic cross-correlation maps (DCCMs) were further plotted at a PCC threshold of 0.4, from matrices representing *|c*_*ij*_ | ≥ 0.4.

Network path analyses were carried out by considering four different combinations of ‘source’ and ‘sink’ residue pairs (start and end nodes) in both Aβ42 and αS: *(1)* N-terminal end and C-terminal end, *(2)* Start of initial helical regions (in the original PDB structures) and end of helix, *(3)* Start of helix 1 and end of helix 1, and *(4)* Start of helix 2 and end of helix 2. Consequently, the optimal path (shortest path) and sub-optimal paths were deduced out of a total of 500 distinct paths constructed for each source and sink pair, using the shortest loopless path algorithm^104^ implemented in Bio3D. Path length (summation over the weights of traversed edges and not representative of an actual geometrical length) distributions with their probability density were compared, which measures the coupling strength between residue pairs. Calculated non-normalized node degeneracies quantify the number of paths traversing each node, highlighting densely connected nodes. In addition, frequently crossed ‘hub’ nodes were identified from measured betweenness centrality (or node centrality) of the number of unique shortest paths crossing each node.

## Supplementary information

Supplementary Information.

## Data Availability

The datasets generated and analysed during the current study are available from the corresponding author on reasonable request.
