# Supplementary material for: Long-range Regulation of Partially Folded Amyloidogenic Peptides
Source: Sci Rep. 2020 May 5;10:7597. doi: 10.1038/s41598-020-64303-x (PMC7200734; doi:10.1038/s41598-020-64303-x)
Supplement: Supplementary file 1 — Supplementary Information. [file 41598_2020_64303_MOESM1_ESM.pdf]

Supplementary Information

Long-range Regulation of Partially Folded Amyloidogenic Peptides

Shayon Bhattacharya,[1] *Liang Xu,*[1] *Damien Thompson*[1,*]

[1]Department of Physics, Bernal Institute, University of Limerick, V94 T9PX, Ireland.

[*]Corresponding author E-mail: damien.thompson@ul.ie; Tel: +353 61237734

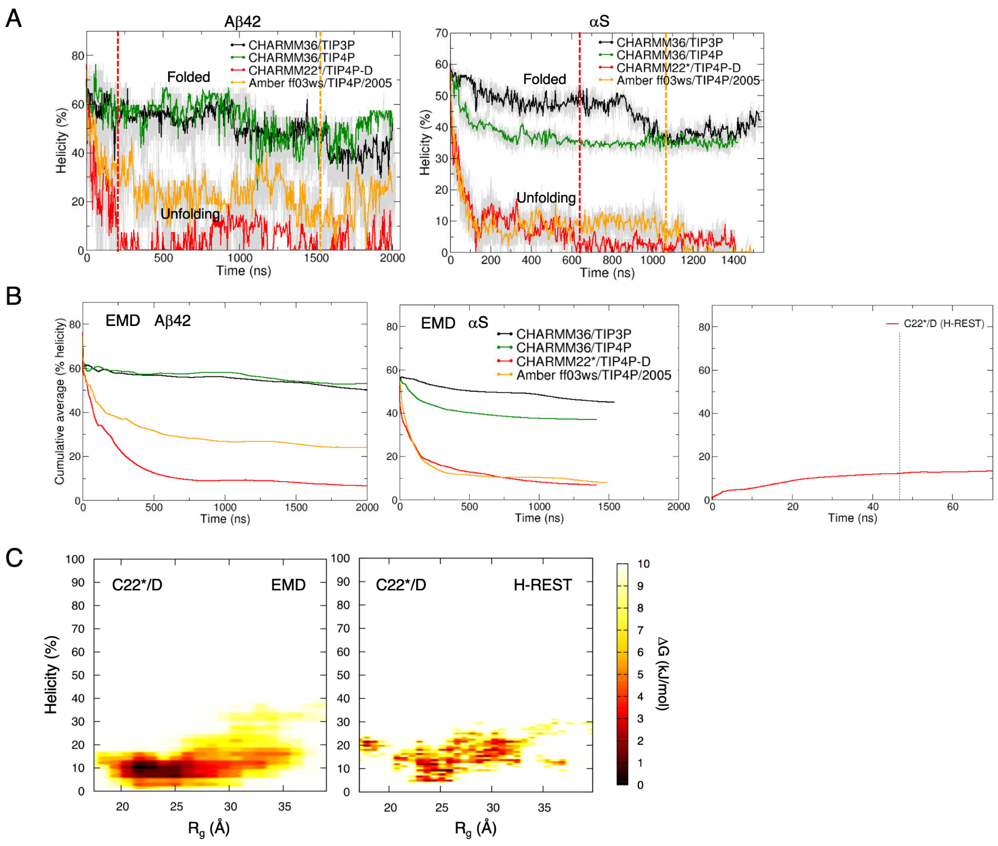

Fig. S1. (A) Temporal evolution of helicity (%) in folded (CHARMM36/TIP3P and CHARMM36/TIP4P), partially folded (CHARMM22*/TIP4P-D and Amber ff03ws/TIP4P/2005, following unfolding and prior to complete disordering), and unfolded (CHARMM22*/TIP4P-D and Amber ff03ws/TIP4P/2005, from completely unfolded to end of simulation) states of Aβ42 and αS. The broken vertical lines represent the time for complete unfolding for CHARMM22*/TIP4P-D (red) and Amber ff03ws/TIP4P/2005 (orange). Running averages over 40 data points are shown. **(B)** Time convergence of cumulative average helical content (%) for the equilibrium MD (EMD) for all the states and one Hamiltonian replica exchange with solute scaling (H-REST) for comparison. **(C)** Comparison of the helical conformational subspaces sampled by EMD and H-REST (αS sampled with CHARMM22*/TIP4P-D is only shown).

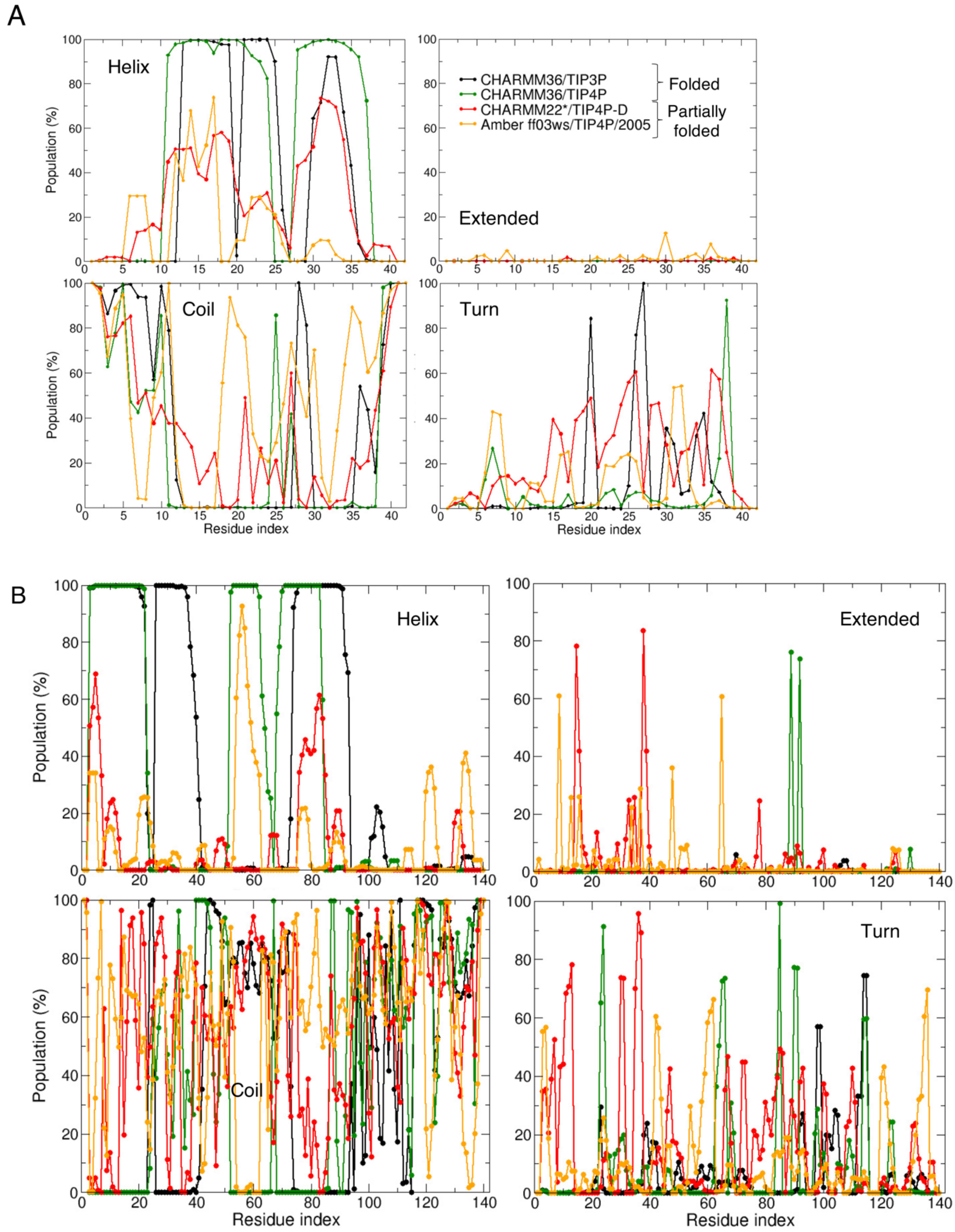

Fig. S2. Time-averaged residue-wise secondary structure probabilities (%) for the last 200 ns of **(A)** Aβ42, and **(B)** αS in their helically folded and partially folded helical states.

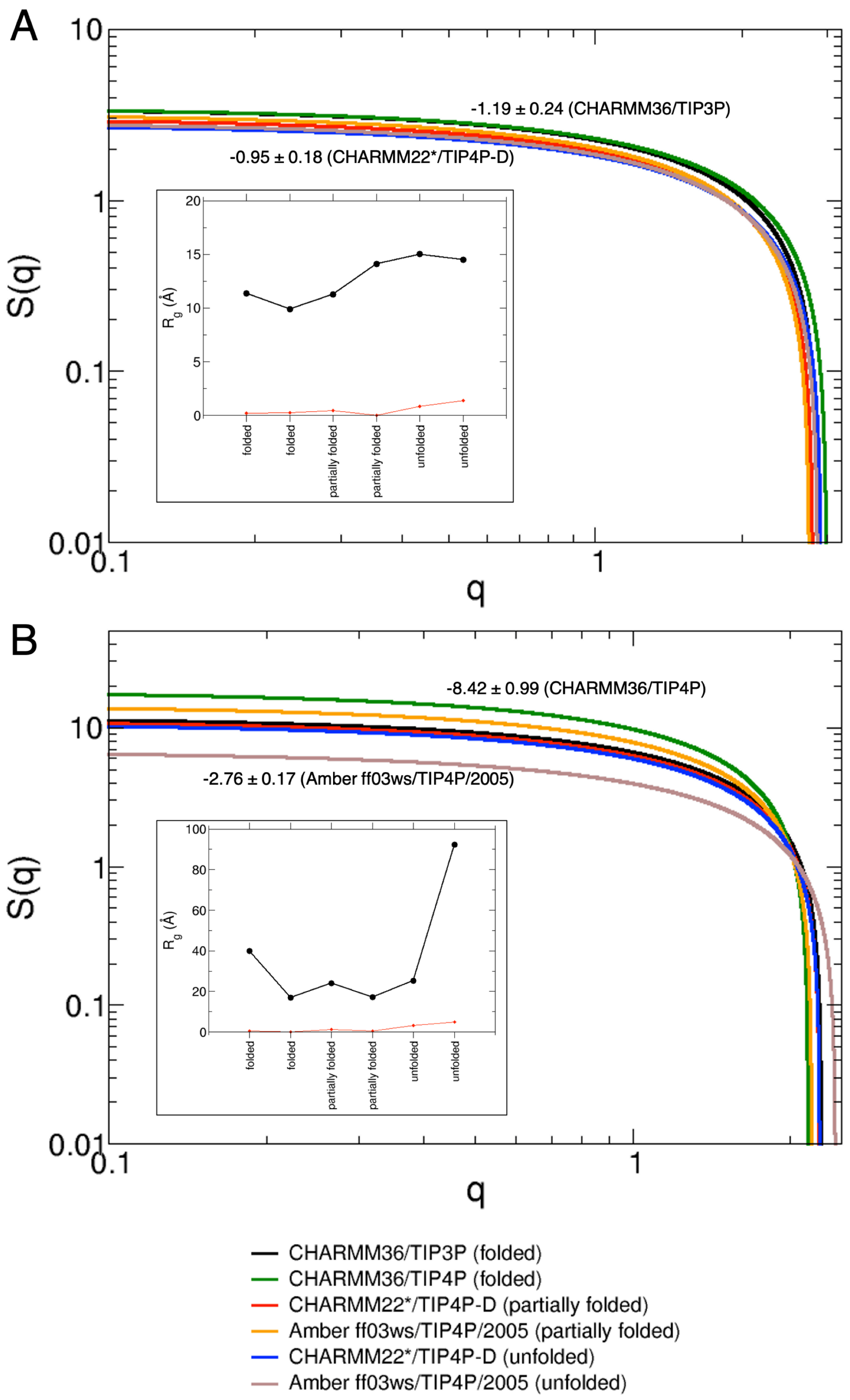

Fig. S3. Structure factor $S(q)$ as a function of the wave vector q for the helically folded, partially folded helical and completely unfolded states of **(A)** Aβ42 and **(B)** αS on a log-log scale. The inset subpanels in the bottom left corner in both panels A and B show the the variation of the R_g (with standard error in red) with the fold propensities from folded to unfolded. The estimates of $-1/\gamma$ (from the slope along with the standard error) for the fully folded and the fully unfolded states of Aβ42 and αS are annotated alongside their corresponding plots.

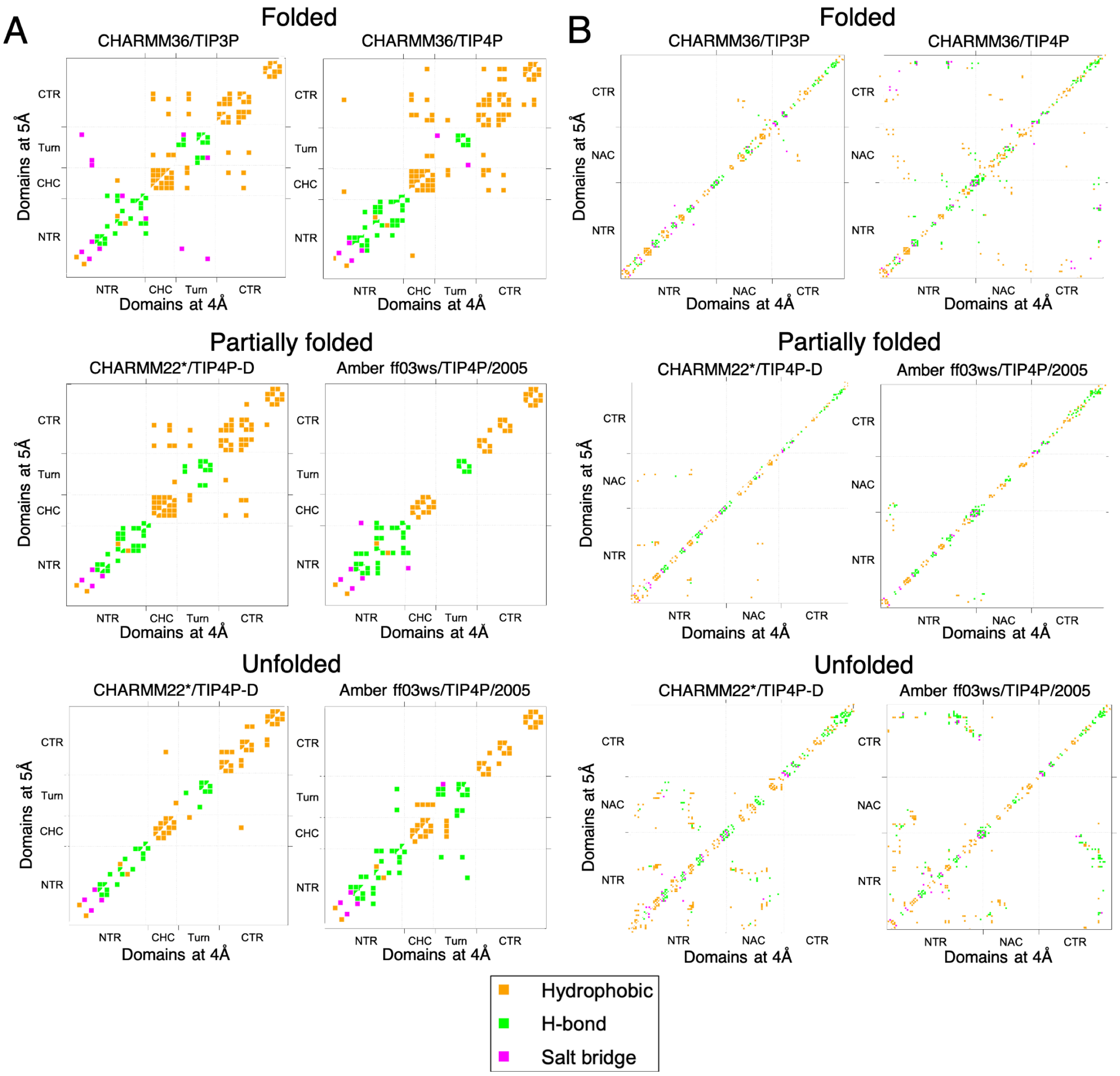

Fig. S4. Inter-domain interaction maps of **(A)** Aβ42 and **(B)** αS computed for their corresponding helically folded, partially folded helical and unfolded states using CONAN[1] tool for analysing tertiary structures. The interactions between domains within 5 Å occupy the upper left triangle, while interactions between domains within 4 Å are in the lower right triangle. Specific interactions in the maps are coloured: orange – hydrophobic, green – hydrogen bond, and purple – salt bridge. The interaction maps were constructed from contacts with probabilities >50%.

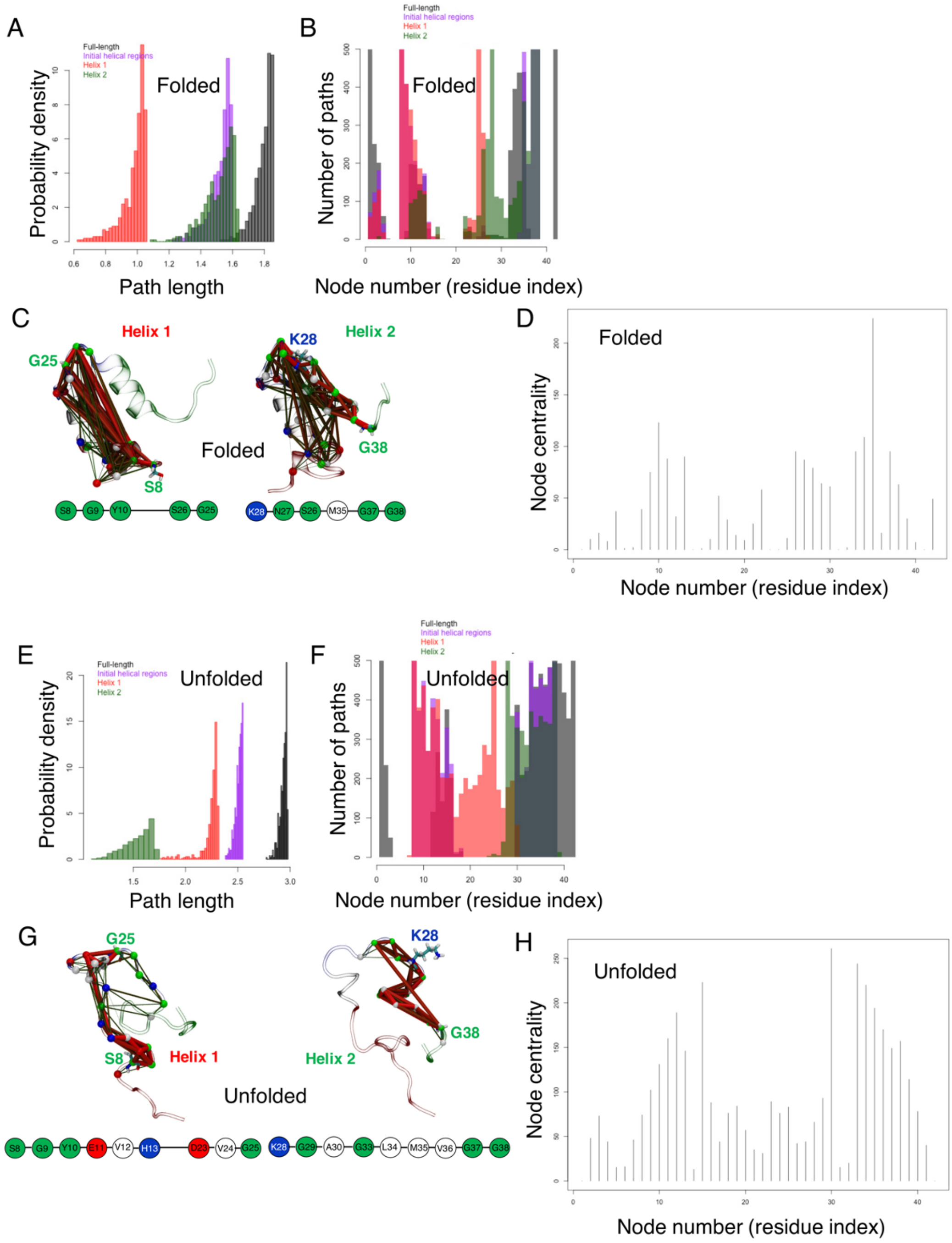

Fig. S5. Dynamic cross-correlation network analyses of the helically folded **(A-D)** and unfolded **(E-H)** states of Aβ42. (**A**) Path length distribution, (**B**) Node degeneracy showing number distribution of paths (out of 500) for each of the source and sink pairs, (**C**) Consensus networks corresponding to regions Helix 1 (source = S8 and sink = G25) and Helix 2 (source = K28 and sink = G38) visualized in VMD with all possible paths, and (**D**) Betweenness centrality distribution, showing the network hubs for the helically folded states. **(E)** Path length distribution, (**F**) Node degeneracy, (**G**) Consensus networks of Helix 1 and Helix 2, and (**H**) Node (betweenness) centrality for the unfolded states. The thickness of the paths indicates how strongly the two residues are correlated. The network structures are overlaid on the most representative folded state, and the corresponding optimal/shortest path of correlated motions is shown below each network.

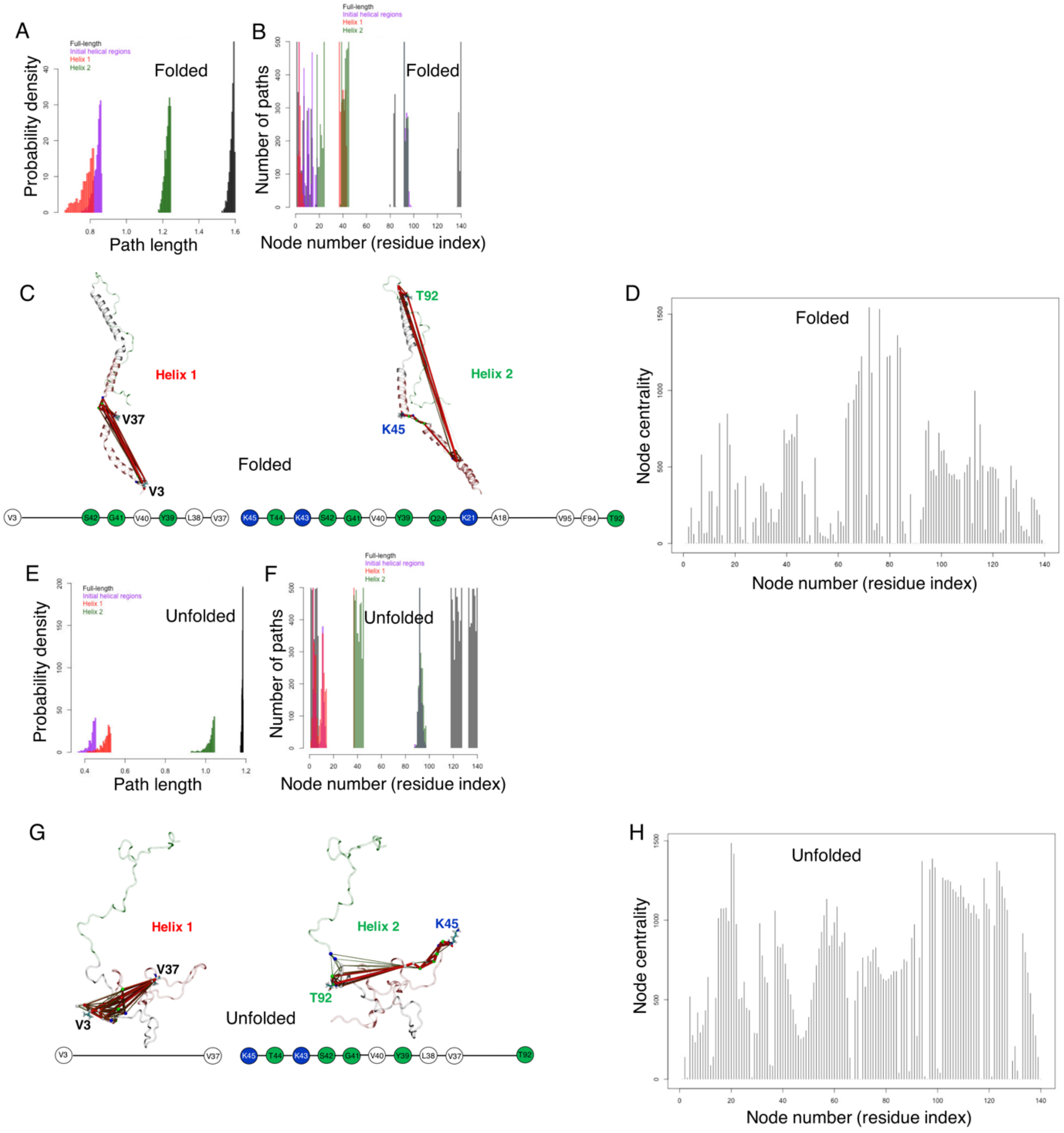

Fig. S6. Dynamic cross-correlation network analyses of the helically folded and unfolded states of αS. (**A**) Path length distribution, (**B**) Node degeneracy, (**C**) Consensus networks corresponding to regions Helix 1 (source = V3 and sink = V37) and Helix 2 (source = K45 and sink = T92), and (**D**) Node centrality for the helically folded states. (**E**) Path length distribution, (**F**) Node degeneracy, (**G**) Consensus networks of Helix 1 and Helix 2, and (**H**) Node centrality for the unfolded states.

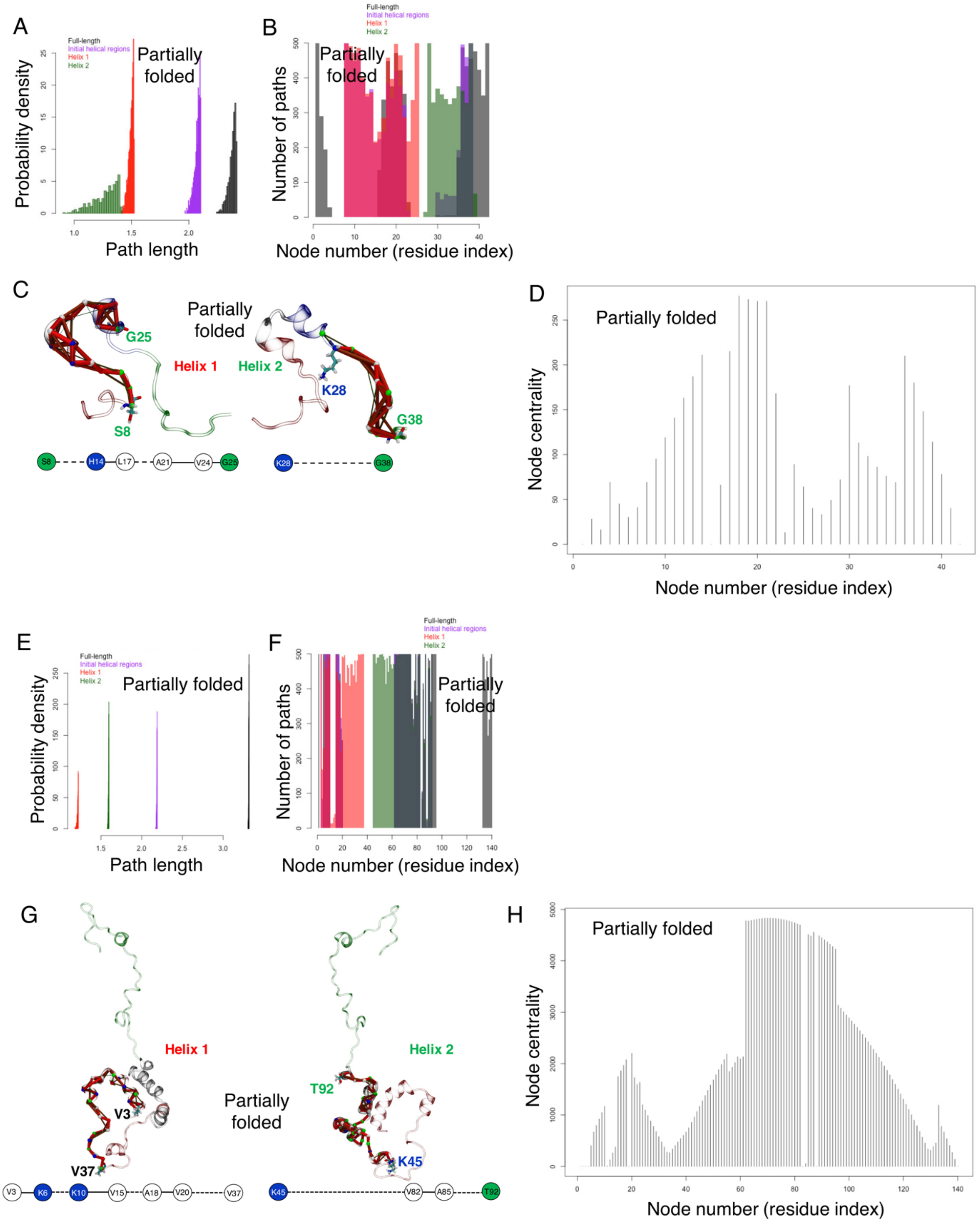

Fig. S7. Dynamic cross-correlation network analyses of the partially folded helical states of Aβ42 and αS. (**A**) Path length distribution, (**B**) Node degeneracy, (**C**) Consensus networks of Helix 1 and Helix 2 (same region as in **Fig. S2**), and **(D)** Betweenness centrality of Aβ42. (**E**) Path length distribution, (**F**) Node degeneracy, (**G**) Consensus networks of Helix 1 and Helix 2 (same region as in **Fig. S3**), and (**H**) Node or betweenness centrality of αS. Broken lines between two nodes represent coupling including all residues in between.

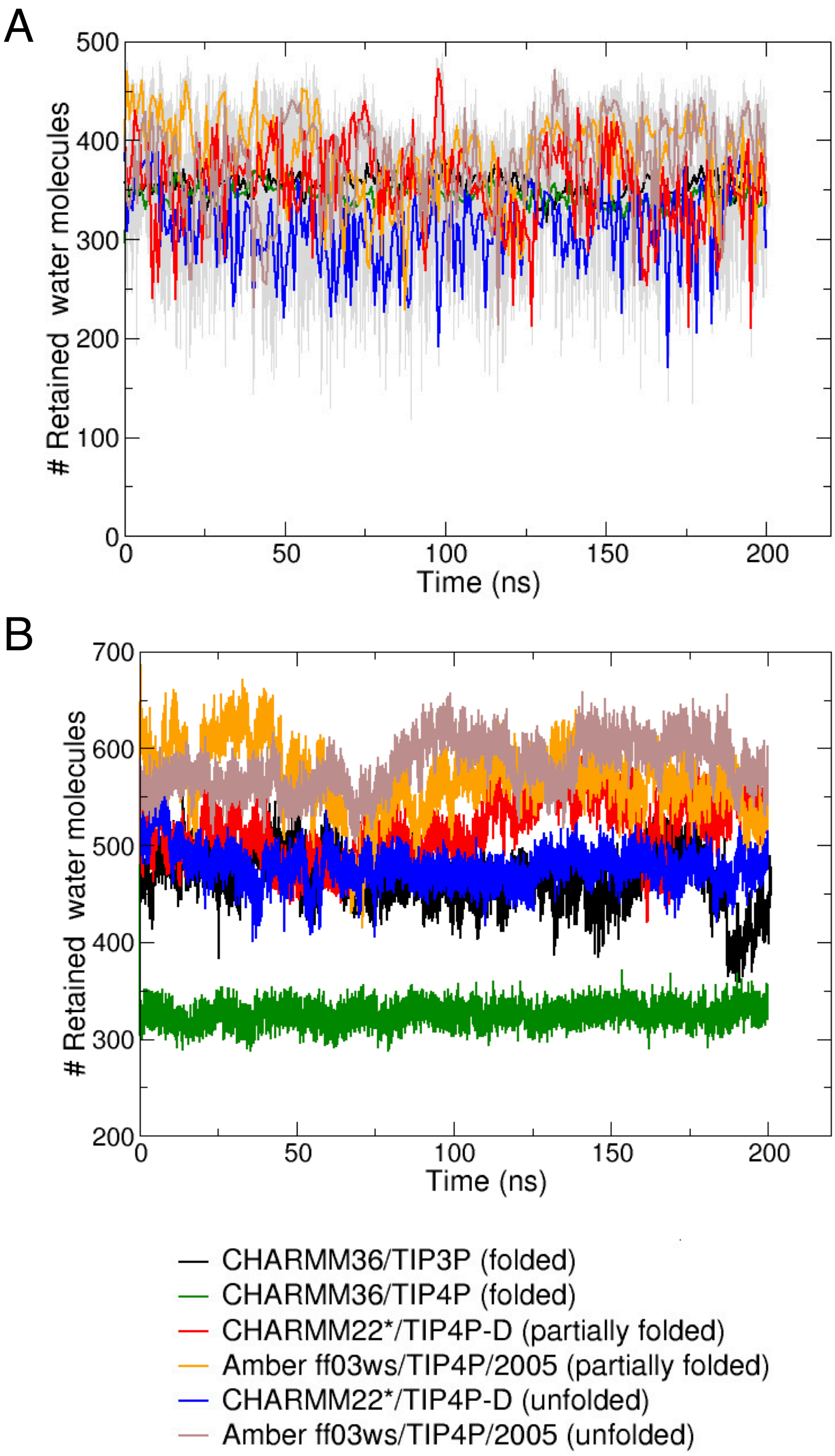

Fig. S8. Number of retained water molecules within a 10 Å hydration layer around the **(A)** CHC of Aβ42 and **(B)** the hydrophobic core (G68 – A78) of NAC in αS. The last 200ns of simulations are shown with averages taken over 20 data points.

1 Mercadante, D., Gräter, F. & Daday, C. CONAN: A Tool to Decode Dynamical Information from Molecular Interaction Maps. *Biophysical Journal* **114**, 1267-1273, doi:10.1016/j.bpj.2018.01.033 (2018).
